# Conducting Benefit-Cost Analysis in Low- and Middle-Income Countries: Introduction to the Special Issue

**DOI:** 10.1017/bca.2019.4

**Published:** 2019-02-27

**Authors:** Lisa A. Robinson, James K. Hammitt, Dean T. Jamison, Damian G. Walker

**Affiliations:** 1 Lisa A. Robinson, Harvard T.H. Chan School of Public Health (Center for Risk Analysis & Center for Health Decision Science), 718 Huntington Avenue, Boston, MA 02115, USA; 2 James K. Hammitt: Harvard T.H. Chan School of Public Health (Center for Risk Analysis & Center for Health Decision Science), 718 Huntington Avenue, Boston, MA 02115, USA and Toulouse School of Economics, Université de Toulouse Capitole, 21 allée de Brienne, 31000 Toulouse, France; 3 Dean T. Jamison: University of California, San Francisco (Global Health Sciences), 550 16th Street, San Francisco, CA 94143, USA; 4 Damian G. Walker: Bill & Melinda Gates Foundation, 500 Fifth Avenue North, Seattle, WA 98109, USA

**Keywords:** benefit-cost analysis, low- and middle-income countries, value per statistical life, D6, H4, I1, Q5

## Abstract

Investing in global health and development requires making difficult choices about what policies to pursue and what level of resources to devote to different initiatives. Methods of economic evaluation are well established and widely used to quantify and compare the impacts of alternative investments. However, if not well conducted and clearly reported, these evaluations can lead to erroneous conclusions. Differences in analytic methods and assumptions can obscure important differences in impacts. To increase the comparability of these evaluations, improve their quality, and expand their use, this special issue includes a series of papers developed to support reference case guidance for benefit-cost analysis. In this introductory article, we discuss the background and context for this work, summarize the process we are following, describe the overall framework, and introduce the articles that follow.

## 1 Introduction and context

Benefit-cost analysis (BCA) and other forms of economic evaluation are powerful tools, encouraging the systematic collection and assessment of the evidence needed to support sound policy decisions. In low- and middle-income countries, where resources are especially scarce and needs are very great, such decisions are particularly difficult and economic evaluations can be very useful. If not well conducted and clearly reported, however, these studies can lead to erroneous conclusions. Differences in analytic methods and assumptions can obscure important differences in policy impacts.

Recognizing these challenges, the Bill & Melinda Gates Foundation (hereafter, the Gates Foundation) is supporting the development of reference case guidelines.^1^ These guidelines are intended to increase the comparability of economic evaluations, improve their quality, and expand their use. The resulting analyses will promote understanding of the difficult trade-offs faced within and across sectors and support decisions by the Gates Foundation, other nongovernmental and governmental organizations, and individuals.

This special issue of the *Journal of Benefit-Cost Analysis* is an important milestone in that process. It includes methods papers that form the basis of the BCA reference case guidance and case studies that illustrate its application. In this article, we discuss the background and context for this work, summarize the process we are following, describe the overall framework, and introduce the articles that follow. We hope this special issue will be useful to readers seeking to learn more about BCA as well as to experienced practitioners.

### 1.1 Background and conceptual framework

The starting point for this work is the International Decision Support Initiative (iDSI) Reference Case (NICE International, 2014; Wilkinson et al., 2016), which was funded by the Gates Foundation to provide general guidance for all types of health-related economic evaluations as well as specific guidance for conducting cost-effectiveness analysis (CEA). The Gates Foundation then funded the project that is the focus of this special issue, which expands the iDSI Reference Case to include BCA.

The iDSI Reference Case concentrates on the use of economic evaluation for health technology assessment, including interventions to prevent or treat particular health conditions primarily within the healthcare system. The goal is to explore the effect of these interventions on health, usually measured as changes in quality-adjusted life years (QALYs) or disability-adjusted life years (DALYs). Both are nonmonetary measures that integrate consideration of health and longevity. In this context, CEA is typically used to determine whether funding a particular intervention is more or less cost-effective than other uses of healthcare resources.

BCA aims to assess the effects of policies on overall welfare rather than solely on health. It uses monetary values to measure the extent to which individuals are willing to exchange their income – which can be spent on other things – for the health and non-health outcomes they will likely experience if a policy is implemented.^2^ The expansion of the reference case to include BCA reflects the goals of the Gates Foundation. While global health continues to be its primary focus, the Gates Foundation also has a strong interest in other sectors such as agriculture, financial services for the poor, water and sanitation, and education. The Gates Foundation expects the application of BCA will inform how it and others allocate their time, money, and other resources both within and across sectors.

The approach for estimating costs is generally the same in CEA and BCA; both rely on estimates of opportunity costs often (but not always) derived from market prices. The expansion of the reference case to encompass BCA thus focuses primarily on approaches for estimating benefits, particularly those that cannot be fully valued using market prices such as changes in the risk of death, illness, or injury.

Whether CEA, BCA, or both should be applied depends on the decision-making context, including the interests of those involved, the nature of the problem to be addressed, and the resources to be reallocated. For example, if the policy question is how to best reallocate the healthcare budget to improve health, then CEA is usually most appropriate. If the policy question is how to best set the healthcare budget, reallocate other government spending, adjust tax policies, or design regulations to increase societal welfare, then BCA is often most appropriate. Because any analytic approach will have advantages and limitations that relate to the data and methods available as well as the underlying assumptions, conducting both CEA and BCA may provide useful insights in many settings.

While the term “benefit-cost analysis” is used generically to refer to any process for weighing harms and improvements, within welfare economics it has a more precise meaning. Conceptually, it is based on two fundamental normative elements. The first is that each individual is the best, or most legitimate, judge of his or her welfare. How individuals’ concerns about other peoples’ wellbeing should be incorporated raises complex issues that are not fully resolved. The second is that the preferred policy is that which maximizes social welfare, measured by summing the effects of policy across individuals. The idea is that concerns about who receives the benefits and who bears the costs should be addressed separately, through policies that directly affect distribution such as the tax and income-support system. Those who are not entirely comfortable with these normative underpinnings may still find the information generated by this framework useful.

As does the iDSI Reference Case, most BCA guidance recommends that economic evaluation should play a major role in the decision-making process but should not be the sole basis for policy decisions. This recommendation in part stems from the need to address normative issues, such as concerns for others’ wellbeing, that may not be adequately captured in these frameworks. Another concern is the need to examine legal, technical, budgetary, and political constraints. Finally, as is the case with any form of evaluation, addressing data gaps and inconsistencies poses many challenges. Analysts must carefully investigate the evidence, identify and assess the effects of uncertainties (including impacts that cannot be quantified), and clearly communicate the implications for decision-making.

### 1.2 Guidance development process

The process used to develop this reference case guidance was designed to encourage extensive involvement from stakeholders, including both BCA practitioners and consumers. The goal is to ensure that the guidance incorporates multiple perspectives and types of expertise, and is both useful and used. We followed a three-phase process.^3^ In the first phase, we explored the potential scope of the guidelines. We reviewed the available guidance and selected analyses, conducted a stakeholder survey, discussed the issues in a public workshop, and solicited comments. We then used the results to set priorities for the subsequent phases.

The articles in this special issue represent the culmination of the second phase. We commissioned a series of 13 papers to develop methodological recommendations and to test them through application to case studies. The drafts were posted online for public comment, discussed in a public workshop, and then revised. Eight of these papers are published in this special issue along with this introductory article.

In the third phase, we are developing guidelines for implementing the BCA reference case. These guidelines will be posted on the project website for public comment then revised and finalized. The final guidelines will be freely accessible online and designed to be easily updated as new research results become available and methods are further developed. We are also drafting recommendations for future work.

Ultimately, the guidelines will provide an overview of the BCA framework, discuss its theoretical foundations, describe how to value specific types of outcomes, examine approaches for assessing uncertainty and non-quantified effects, and discuss the presentation of the results. They will incorporate recommendations from the methods papers included in this special issue and use the case studies to illustrate their application.

## 2 General framework

To frame the methodological guidance and case studies included in this special issue, we begin by discussing the major BCA components. While this framework will be familiar to most readers of this journal, translating it into reference case guidance requires addressing several concerns as described below.

**Figure 1:**
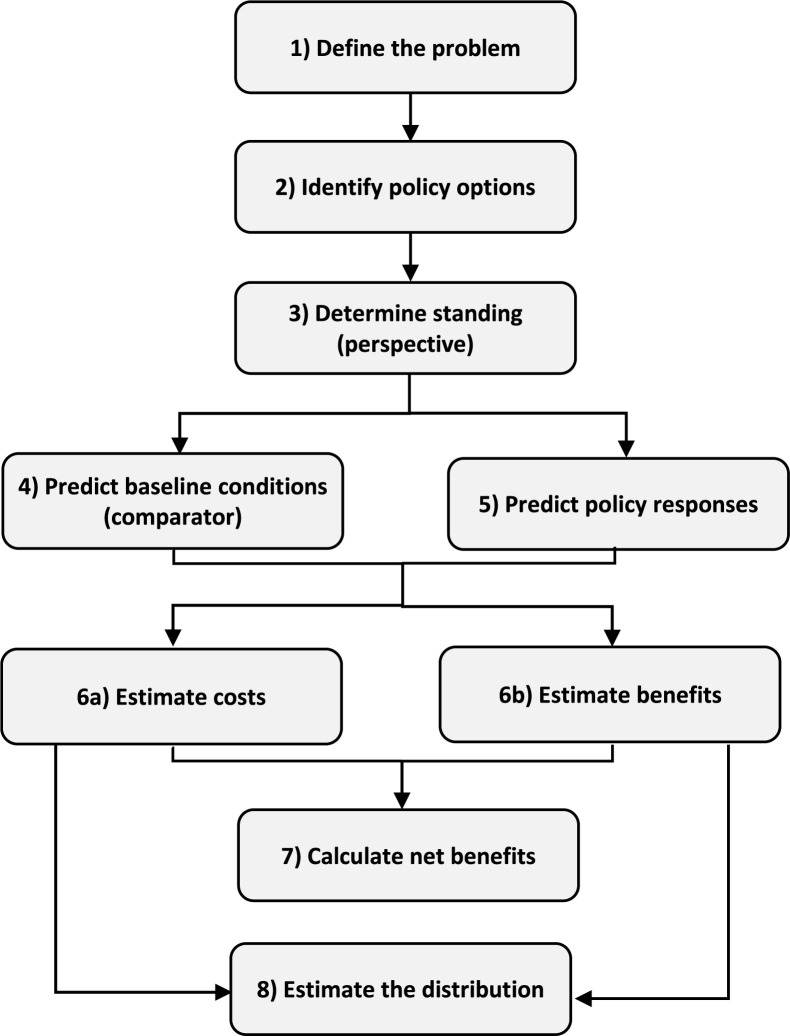
BCA Components.

As conventionally conducted, BCA consists of seven basic components; distributional analysis is a desirable eighth component, as illustrated in Figure 1. While shown as if it were a sequential process, in reality these steps are iterative. As analysts acquire additional information and review their preliminary findings, they often revise earlier components to reflect improved understanding of the issues. Each of these steps requires consideration of uncertainty as well as non-quantified effects.

As suggested by Figure 1, methods for conducting BCA are most well-developed and widely used to assess options for addressing a particular problem. In other words, these methods focus largely on analyzing policies that can be appropriately evaluated within a microeconomic framework and are not expected to significantly affect prices or the economy at large. Although many of the concepts we discuss are applicable within a macroeconomic framework, that is not our primary concern. This means we do not address issues such as extending national income and product accounts to include nonmarket welfare measures (see, for example, Usher, 1973; Jamison et al., 2013) or assessing the total economic damages or burden attributable to specific health conditions, causes, or risk factors (see, for example, Landrigan et al., 2018).^4^

We briefly introduce each component below and discuss some general implementation issues. For simplicity, this overview assumes the BCA is conducted from a prospective, *ex ante* perspective, before the policy is implemented. It may also be conducted from a retrospective, *ex post* perspective, after the impacts of the policy have materialized, to compare the results to what would likely have occurred in the absence of the policy.

In this section, we cite the methods papers drafted to support the development of the reference case guidance, including both articles included in this special issue and additional working papers posted on the project website. The implementation of selected aspects of this framework is illustrated by the four case studies in this special issue (Cropper et al., 2019; Pradhan & Jamison, 2019; Wilkinson et al., 2019; Wong & Radin, 2019), as well as by two additional case studies posted on our website (Neumann et al., 2018; Radin et al., 2019).

**(1) Define the problem**: BCA is often motivated by a specific problem or policy goal, which may be identified by the analyst, a policymaker, or others. As illustrated by the case studies, the problem may involve more effectively controlling tuberculosis, reducing poor nutrition, decreasing air pollution, improving educational attainment, or other goals. It may also be motivated by interest in prioritizing spending across interventions in different policy areas. Whatever the policy goal, the analysis should be comprehensive in including all significant consequences.

**(2) Identify policy options**: While many studies assess only a single option for addressing the problem, considering several reasonable alternatives is preferable. Evaluating only one option can lead decision-makers to ignore others that may be more cost-beneficial.

**(3) Determine who has standing (perspective)**: Standing refers to identifying whose benefits and costs will be counted. The analysis may, for example, consider impacts on only those who reside or work in a specific country or region, or may address international impacts. This concept is related to that of “perspective” in CEA. For example, a CEA may be conducted from the societal perspective, in which case all impacts are included, or from the perspective of the healthcare sector, in which case only the impacts on that sector are considered.

When the question of standing or perspective raises difficult issues, it is often useful to report the results at different levels of aggregation rather than trying to fully resolve these issues prior to conducting the analysis. For example, the results could be reported for a specific region, for the country as a whole, and at the global level, or for the healthcare system alone and for society at large.

**(4) Predict baseline conditions (comparator)**: Each policy option is typically compared to a “no action” baseline that reflects predicted future conditions in the absence of the policy, although other comparators may at times be used. The baseline should reflect expected changes in the status quo. For example, the health of the population and its size and composition may be changing, and the economy may be evolving, in ways that will affect the incremental impact of a policy.

**(5) Predict policy responses**: This component involves predicting the impacts of each option in comparison to the baseline or other comparator. One challenge is ensuring that changes likely to occur under the baseline are not inappropriately attributed to the policy; another is understanding the causal pathway that links the policy to the outcomes of concern. The goal is to represent the policy impacts as realistically as possible, taking into account real-world behavior.

These impacts should be described both qualitatively and quantitatively, comparing predictions under baseline conditions to predictions under the policy. Related measures should include, at minimum, estimates of the expected number of individuals and entities affected in each year, along with information on their characteristics. For policies that affect health and longevity, the expected number of deaths and cases of illness, injuries, or other disabilities averted in each year should also be reported.

**(6) Estimate costs and benefits**: Whether a consequence is categorized as a “cost” or “benefit” is arbitrary and varies across BCAs. However, consistent categorization is essential for comparability across analyses. As long as the sign is correct (positive or negative), the categorization of an impact as a cost or a benefit will not affect the estimate of net benefits, but will affect the ratio. If categorized inconsistently, benefit-cost ratios, total costs, and total benefits cannot be meaningfully compared.

One intuitively appealing option is to distinguish between inputs and outputs. Under this scheme, costs are the required inputs or investments needed to implement and operate the policy – including real resource expenditures such as labor and materials, regardless of whether these are incurred by government, private or nonprofit organizations, or individuals. Benefits are then the outputs or outcomes of the policy; i.e., changes in welfare such as reduced risk of death, illness, or injury.

Under this framework, counterbalancing effects are assigned to the same category as the impact they offset. For example, “costs” might include expenditures on improved technology as well as any cost savings that result from its use; “benefits” might include the reduction in disease incidence as well as any offsetting risks, such as adverse reactions to vaccines.

This project does not address the estimation of costs in detail. That topic is covered by the iDSI Reference Case we supplement (NICE International, 2014; Wilkinson et al., 2016) as well as by the work of the Global Health Cost Consortium (Vassall et al., 2017). Standard CEA and BCA texts also provide more information on cost estimation (e.g., Drummond et al., 2015; Boardman et al., 2018).

Our focus is largely on the estimation of benefits, particularly those that cannot be fully valued using market prices. For example, as discussed in Robinson and Hammitt (2018) and Robinson et al. (2019), valuing changes in health and longevity generally requires the use of revealed- or stated-preference methods. Revealed-preference methods estimate the value of nonmarket outcomes based on the prices paid for related market goods, while stated-preference methods estimate these values based on survey data. In addition, Skinner et al. (2019) discuss the value of increased financial risk protection associated with health insurance.

Another area that often requires the use of nonmarket valuation methods is changes in time use. For example, a vaccination program may require that individuals travel to a healthcare center, decreasing the time available for other activities. A program providing cleaner water could either increase or decrease the time required to travel to the water source. While compensation rates are often used to value changes in the use of paid time, these activities frequently involve changes in the use of unpaid time. Such changes may be categorized as either a cost or a benefit, depending on whether the change contributes to implementation of a policy (a cost) or is among its outcomes (a benefit). Whittington and Cook (2019) discuss the appropriate valuation of changes in time use in more detail.

**(7) Calculate net benefits**: The final step in the BCA involves comparing costs and benefits. As part of this calculation, future year impacts are discounted to reflect time preferences as well as the opportunity costs of investments made in different periods, as discussed in Claxton et al. (2019). This discounting reflects the general desire to receive benefits early and to defer costs. The monetary values of benefits and costs should be discounted at the same rate.

The results are often reported as net benefits (benefits minus costs). Benefit-cost ratios or the internal rate of return (IRR) may also be used, but must be constructed and interpreted with care. As noted above, the benefit-cost ratio depends on how components are classified as benefits or costs. The IRR, which is the discount rate at which the present value of net benefits is zero, may not be unique if net benefits change sign more than once over time.

Because benefit-cost ratios and IRRs are not sensitive to scale, they can be misleading if used alone. The magnitude of the impacts must also be considered. Two policies with identical benefit-cost ratios or IRRs may yield substantially different net benefits. For example, a policy with $1,000 in benefits and $100 in costs and a policy with $1,000,000 in benefits and $100,000 in costs both have benefit-cost ratios of 10, but the latter policy leads to substantially larger improvements in welfare. Similarly, if the costs of these policies occur in the current year and the benefits occur 10 years later, they have the same IRR (29 %) but the second policy has the larger present value if the discount rate is smaller than the IRR. Thus, net benefits should always be reported along with these summary measures.

**(8) Estimate the distribution of impacts**: While often considered to be outside the BCA framework, the distribution of impacts across a population is frequently important to decision-makers and other stakeholders. At minimum, analysts should provide descriptive information on how the costs, benefits, and net benefits are likely to be allocated across income and other groups (Robinson et al., 2018).

Each of the above components requires appropriate consideration of uncertainty, including non-quantified effects. In summarizing the results, analysts should address the extent to which these uncertainties affect the likelihood that a particular policy yields positive net benefits and the relative ranking of the policy options.

Because analytic resources are limited, the ideal analysis will not assess all policy options nor quantify all outcomes with equal precision. In some cases, the cost of analyzing a particular option or quantifying a specific outcome will be greater than the likely benefit of assessing it, given its importance for decision-making. In other words, the analysis may not sufficiently improve the basis for decision-making to pass an implicit benefit-cost or value-of-information test. Conversely, options and outcomes that are important for decision-making should receive substantial attention.

To implement the BCA framework, analysts should begin by listing all potential costs, benefits, and other impacts, then use screening analysis to identify the impacts most in need of further investigation. Screening analysis relies on easily accessible information and simple assumptions to provide preliminary insights into the direction and magnitude of effects. For example, upper-bound estimates of parameter values can be used to determine whether particular impacts may be significant. Screening aids analysts in justifying decisions to exclude impacts from more detailed assessment and in determining where additional research is most needed to reduce uncertainty. It also provides data that can be used to indicate the rough magnitude of impacts that are not assessed in detail.

## 3 Articles in this special issue

This special issue includes four papers that discuss methods for addressing specific analytic components and four that test the application of these methods to specific case studies. We describe each paper below.

**“Valuing Mortality Risk Reductions in Global Benefit-Cost Analysis,” by Lisa A. Robinson, James K. Hammitt, and Lucy O’Keeffe**. The value of mortality risk reductions (the value per statistical life, VSL) is an important determinant of the benefits of many public health, safety, and environmental policies. While these values are relatively well studied in high-income countries, less is known about the values held by the populations of low- and middle-income countries. This paper reviews the literature and recommends an approach for conducting a standardized sensitivity analysis to facilitate comparison to other studies and to explore the effects of uncertainties. The authors recommend extrapolating from estimated VSL in high-income countries, combining different base estimates and assumptions about the degree to which VSL changes as income decreases. The findings emphasize the need for more research to address the uncertainty in these estimates.

**“Valuing Changes in Time Use in Low- and Middle-Income Countries,” by Dale Whittington and Joseph Cook**. The value of changes in time use is frequently a critical component of economic analyses of public health and development policies. Often these policies change how individuals use their time outside of the formal labor market. This paper reviews the literature and recommends estimating values in low- and middle-income countries by using common defaults from the literature to adjust the after-tax wage rate of the individuals affected. The authors also describe a relatively simple approach for conducting new stated-preference research to estimate these values for the relevant population.

**“Accounting for Timing when Assessing Health-Related Projects,” by Karl Claxton, Miqdad Asaria, Collins Chansa, Julian Jamison, James Lomas, Jessica Ochalek, and Mike Paulden**. The appropriate discounting of future impacts poses a number of complex challenges when assessing health-related policies. These include understanding the relationship of the approach to the policy context as well as addressing gaps and inconsistencies in the empirical research. The authors discuss the overall framework for discounting and offer guidance on its application in different contexts. They consider the use of discounting to convert time streams of different impacts into equivalent health, healthcare cost, and consumption effects.

**“Valuing Protection against Health-Related Financial Risks,” by Jonathan Skinner, Kalipso Chalkidou, and Dean T. Jamison**. While there is strong interest in expanding health insurance coverage (broadly construed to include social insurance and public finance of health systems), such expansion raises difficult issues related to understanding impacts on individuals’ financial wellbeing as well as their health. This paper provides a framework for assessing three distinct financial benefits: pooling the risk of unexpected medical expenditures between healthy and sick households, redistributing resources from high- to low-income recipients, and smoothing consumption over time. The authors illustrate the application of the framework and provide guidance for its application.

**“Comparing the Application of CEA and BCA to Tuberculosis Control Interventions in South Africa,” by Thomas Wilkinson, Fiammetta Bozzani, Anna Vassall, Michelle Remme, and Edina Sinanovic**. Achieving ambitious targets for addressing the tuberculosis epidemic globally requires understanding the impacts of competing interventions. This case study compares the implementation of CEA and BCA to assess the effects of selected alternatives, converting estimates of the change in DALYs to monetary estimates of the value of reduced mortality and morbidity risks. The authors find that the CEA and the BCA come to somewhat different conclusions about the relative merits of different options in the South African context, and explore the implications of the analytic approach and the interpretation of the results.

**“Benefit-Cost Analysis of a Package of Early Childhood Interventions to Improve Nutrition in Haiti,” by Brad Wong and Mark Radin**. Poor nutrition is a major issue in many low- and middle-income countries that requires the combination of several strategies to be effectively addressed. This case study considers a package of ten interventions that target pregnant women and children in Haiti, such as salt iodization and nutrient supplementation. It considers the impacts on health, longevity, and lifetime productivity, testing the effects of different methodological choices. The authors conclude that the net benefits are substantial, but that other policies may be more cost-effective.

**“Applying Benefit-Cost Analysis to Air Pollution Control in the Indian Power Sector,” by Maureen Cropper, Sarath Guttikunda, Puja Jawahar, Zachary Lazri, Kabir Malik, Xiao-Peng Song, and Xinlu Yao**. Air pollution is a persistent and well-established public health problem to which emissions from coal-fired power plants significantly contribute. This case study evaluates the impacts of retrofitting plants in India to reduce emissions, testing the effects of alternative approaches for valuing mortality risk reductions. The authors find that the net benefits of pollution control vary widely by location, and that the number of plants for which installation of controls is cost-beneficial depends on the VSL used.

**“Standardized Sensitivity Analysis in BCA: An Education Case Study,” Elina Pradhan and Dean T. Jamison**. BCAs of educational enhancements in low- and middle-income countries typically focus primarily on the impacts on future wages. Strong evidence suggests that education is also likely to increase the longevity of both students and their young children. This case study explores variation in the net benefits of an additional year of schooling in lower–middle-income countries. Net benefits depend on the value of mortality risk reductions, including adjustments for income and age. The authors find that including the value of mortality risk reductions substantially increases the estimated net benefits of schooling.

In sum, these papers propose and test the application of methods for BCA that can be feasibly implemented based on the data and research now available. They also illustrate the need for substantial additional work. In particular, relatively little is known about the preferences of low- and middle-income populations for improvements in health, longevity, and other aspects of wellbeing. Additional research is needed to increase understanding of these preferences and better tailor the analysis to the population of concern.

## Footnotes

1 In guidance for cost-effectiveness analyses of health and medical interventions, the term “reference case” is often used to refer to a standard set of practices that analysts should follow to improve the comparability and quality of the analyses. In BCA, the terms “guidance” or “guidelines” are often used to refer to the same types of best practice standards. Thus, we use both terms in this article.

2 We use the term “policy” as a generic term to include projects, programs, interventions, and other actions that affect the wellbeing of multiple individuals in a society.

3 More information on this project, including related reports, working papers, and workshop materials, is available on our website: https://sites.sph.harvard.edu/bcaguidelines/.

4 Strzepek et al. (2018) and Neumann et al. (2018) discuss the use of computable general equilibrium models to estimate economy-wide impacts.
